# Applications Based on Service-Oriented Architecture (SOA) in the Field of Home Healthcare

**DOI:** 10.3390/s17081703

**Published:** 2017-07-25

**Authors:** Karen Avila, Paul Sanmartin, Daladier Jabba, Miguel Jimeno

**Affiliations:** 1 Departamento de Ingeniería de Sistemas, Universidad del Norte, Barranquilla 080001, Colombia; djabba@uninorte.edu.co (D.J.); majimeno@uninorte.edu.co (M.J.); 2 Departamento de Ingeniería de Sistemas, Universidad Simón Bolivar, Barranquilla 080001, Colombia

**Keywords:** SOA, telemedicine, healthcare, IoT, body sensor networks

## Abstract

This article makes a literature review of applications developed in the health industry which are focused on patient care from home and implement a service-oriented (SOA) design in architecture. Throughout this work, the applicability of the concept of Internet of Things (IoT) in the field of telemedicine and health care in general is evaluated. It also performs an introduction to the concept of SOA and its main features, making a small emphasis on safety aspects. As a central theme, the description of different solutions that can be found in the health industry is developed, especially those whose goal is health care at home; the main component of these solutions are body sensor networks. Finally, an analysis of the literature from the perspectives of functionalities, security implementation and semantic interoperability is made to have a better understanding of what has been done and which are probable research paths to be studied in the future.

## 1. Introduction

Remote healthcare has boomed in recent years, as different mechanisms of remote patient health have been developed. When we refer to telemedicine, we refer to the provision of medical services remotely through technology. Within these services, we can include diagnostic or consultations with specialists; this process occurs mainly in areas of difficult access. This is where the healthcare specialist providing services remotely uses specialized equipment and the help of a local healthcare assistant who will meet the patient and should be able to interpret observations of the specialist and forward it to the patient accurately.

The Internet of Things (IoT) was proposed by Kevin Ashton in the Auto-ID Center at MIT in 1999, where investigations were conducted in the field of network radio frequency identification (RFID) and sensor technologies [1]. The concept of IoT is basically that everyday objects can have an Internet connection at any time and place; this can be achieved through the implementation of sensors. The theory of IoT can fit perfectly in the field of health. The different IoT solutions/applications in the area of health can be sectored in: telemedicine, emergency medication, social networking for health, home health, intelligent pharmaceutical packages and biomedical devices [2].

When we talk about home healthcare, we refer to the supervision of the patient from the comfort of their home, through sensors installed in their body or in their house, which are capable of carrying a registration of patient behavior, or send an alert to health specialist if any anomalous behavior occurs. Many applications developed in this topic are aimed at helping the elderly to live independently at home, for example to help a patient with Alzheimer´s to improve their autonomy.

Sensors for health monitoring at home can be divided into two categories: the sensors that measure physiological parameters, and sensors measuring environmental data [3]. The parameters measured by the physiological sensors can be: heart rate, breathing rate, oxygen saturation (SpO2), body temperature, blood sugar, etc. Environmental sensors can measure parameters related to the intensity of illumination, patient location within the home, home appliances status (on/off), etc. A physiological sensor network is called Body Area Sensor Network (BASN); in this network, a group of wearable sensors are deployed on the body of a person. In general, the sensor nodes have several limitations such as data aggregation, security, heterogeneity of the sensors’ networks, power consumption, among others [4,5]. Security in this type of applications is important, as the type of data being processed and stored is very sensitive, which is why we will consider this topic when selecting the final list of papers. Another important aspect of sensor networks, and also in body sensor networks (BSN), is the fact that it is important to maintain semantic interoperability, i.e., maintain the meaning of the data in all layers of the network architecture. Semantic interoperability in healthcare solutions based on SOA allows standardization and the possibility to change just the services that deal with standards in the case is needed. We will also cover in this work how IoT solutions for healthcare have addressed the problem of semantic interoperability.

Remote patient monitoring systems that monitor the patient and stores the recorded data can employ a service-oriented architecture (SOA) to create federated, distributed and scalable architectures. Web-based management systems are used to enable integration and interoperability between distributed network of sensors and a wireless Internet [6]. The functionality of a service-oriented architecture is the exposure of one or more interfaces through a given program. These interfaces define different methods that are accessible through the network. These interfaces are referred to as “services” [7]. This concept of services should not be confused with the related concept in the field of business administration. WSDL, SOAP (Simple Object Access Protocol), BPEL (Business Process Execution Language), WS-CDL (Web Service Choreography Description Language), UDDI (Universal Description, Discovery and Integration) and RESTful are the best known standards in the use of SOA [8].

In this article, we will see different developed systems in the context of home healthcare based on a service-oriented architecture. The main contributions of this paper are: (1) a review of functionalities offered by healthcare applications based on SOA; (2) an in depth analysis of security implementations in SOA health applications; and (3) a review of semantic operability approaches for healthcare solutions based on SOA.

This review is organized as follows: Section 2 details how the references were selected for this article, databases consulted, keywords used, etc. Section 3 details the concept of SOA and its main features. Section 4 details applications developed in the health area using the SOA architecture. Section 5 performs a comparative analysis of the different applications selected for this review, which covers the measurement variables used, i.e., ECG, PPG, blood pressure, etc. Section 6 performs a comparative analysis of the security mechanisms used in the revised solutions. Finally, Section 7 highlights the conclusions of this paper.

## 2. Methods

### 2.1. Literature Search

This review was conducted through research in several databases. The methodology used for the preparation of this article was developed as follows. Three databases were explored: IEEE, Science Direct, and Scopus. The keywords used and the results obtained in each of the searches are shown in Table 1. It is noteworthy that in the related results filtering was not performed.

### 2.2. Selection Criteria

For the final selection of articles to be included, we took into account the approach that was given to this article. This article deals with applications based on telemedicine, specifically those that implement solutions for patient care at home; these applications had to implement a service-oriented architecture. In general, the articles that supported basic concepts such as telemedicine, service-oriented architecture, security, etc. were considered.

Thus, the search was divided into four categories: previous concepts, applications with SOA architecture in health industry, and SOA security aspects. After inspection of the most relevant articles according to their abstracts and conclusions, 50 articles were selected. For articles whose proposal was the implementation of a computer application, only those published after 2005 were considered.

## 3. Service Oriented Architecture: SOA

SOA promises to close the gap between industrial devices and enterprise applications [9,10,11]. The functionality of a model of a service-oriented architecture is the exposure of one or more interfaces through a given program. These interfaces define different methods that are accessible through the network. These interfaces are referred to as “services” [7,12]. Those who consume these services are called service consumers, and those who provide them are called service providers. Each one of these services is independent, and includes the business logic and data associated with offered service. The services can become interconnected with each other, thus improving the potential of the architecture. For that purpose, architectures need to use a set of rules or protocols to define the message format. Those protocols define a policy of data exchange. SOA is a term that represents a model in which the logic is broken down into different and smaller units. These units may be distributed, and together form part of an automated business logic. A significant advantage in the implementation of SOA is agility in responding to changing business requirements [13]. Interoperability and easier maintenance become an advantage when SOA is implemented correctly, however, if we want to implement a SOA-based architecture we must consider the preservation of its main objectives: the infrastructure must support flexibility, heterogeneity, distributed development and management [13,14].

An important aspect of SOA is the separation of the interface or service (the what) from their implementation or content (the how). The interface provides service identification, while the content provides business logic [15]. Zimmermann et al. [16] suggest three levels of abstraction in SOA:

- Operations: Units of functions operating on received data, having specific interfaces and returning structured responses.
- Services: Logical groupings of operations.
- Business processes: Actions or activities to perform specific business goals by invoking multiple services.

The types of services offered by SOA can be categorized as follows [17]:

- Infrastructure services: Includes security, management and monitoring.
- Business-neutral services: Includes service brokers and notification, scheduling and workflow services.
- Business services: Includes services based on the business logic.

According to [18], a SOA architecture framework may be considered as a seven-layer architecture.

- Layer 1: Operational systems layer; this layer integrates existing systems using SOA integration techniques.
- Layer 2: Enterprise components layer is responsible for realizing functionality and maintaining the quality of service (QoS) of the exposed services.
- Layer 3: Services layer; in this layer, the services selected to be consumed are located.
- Layer 4: Business processes layer, which defines the services exposed in layer 3.
- Layer 5: Access layer; it is created to provide end-to-end solutions to compositions of services.
- Layer 6: Integration layer; it allows the integration of different services.
- Layer 7: Management and security; it is responsible to monitor, manage and maintain security.

### Security in SOA Applications

Security is one of the most important challenges in WSN [4]. The use of online services can expose the eHealth to security threats, similar to any other online application. It becomes a necessity that the identities of legitimate user’s eHealth need to be checked carefully before granting access privileges [19].

SOA increases the number of access points for enterprise systems, increasing also the vulnerability of the site, because many of these points are exposed to the Internet [20,21,22]. Enterprise-level security has changed with technological advances, and has always wanted to maintain a “wall” between what is to be protected and those who have access on this with the aim of preventing access to intruders or unauthorized personnel.

Not only the architecture must be protected from those who access the system over the Internet, but it also must be protected from those who have access through the intranet, because internal networks can be accessed from unsafe physical points within the organization [20]. The progress of the SOA implementation is directly proportional to security risks generated by it.

## 4. Revised SOA Applications

### 4.1. SOA Application in Healthcare Industry

Entities related to the provision of health services (laboratories, pharmacies, clinics, hospitals, etc.), whether public or private, have their own information system. However, in some cases, a patient has contact with more than one establishment in a time range. Under this argument, integration of the multiple information systems the patient has contact with is necessary to be able to handle a general and updated medical history through the communication between different healthcare providers. An advancement of this is the integration of hospital information systems (HIS).

Integration of HIS is one of the most important priorities of the healthcare industry [13]. Information systems belonging to the medical network are inherently heterogeneous, even within the same hospital multiple systems can coexist (e.g., medicine stock software and medical appointment software). Such systems are also classified as heterogeneous. Setareh et al. [13] presents a cloud computing model for integration of hospital information systems which is based on service oriented architecture.

Another area where the healthcare industry is strongly interested is the telemedicine, which refers to the remotely provision of medical services through technology. This process occurs mainly in areas of difficult access. Healthcare professionals provide services remotely using specialized equipment and a local assistant who performs a face-to-face appointment with the patient. The assistant should accurately interpret and forward results to the patient. In [23], the authors presented a project centered on the concepts of ubiquitous healthcare services provided to the patients in rural or remote areas from distant hospitals. In the developed application, a SOA involves mobile client, web services, security agents, business logic layer, data access layer and a database in secured environments.

Another field of action for the development of applications in the healthcare industry is the provision of medical service itself, i.e., when direct contact between a specialist or nurse and the patient is given. We can think of a system that records data collected from the patient during medical consultation. This can be useful when the specialist wants to check the evolution of the medical condition of a patient, which has been tracked with the necessary devices. Similarly, through this type of system, other specialists can access data remotely to support diagnoses. In [24], the authors developed a SOA-based prototype that provides interoperability between different computer platforms, which allowed communication between the clinic, pharmacy, and a network of sensors that capture patient data (i.e., blood pressure). In [25], the authors present an implementation of a web-based medical image annotation system for tele-radiology, and through SOA, the framework is able to support collaboration among radiologists.

### 4.2. SOA Applications in Home Healthcare

Today, the need for an optimal management of the patient's health is very high, and it becomes more critical if management is done remotely while the patient is at their home [26]. Trinugroho et al. [27] proposed the idea of adopting SOA to support eHealth services integration in a smart home environment. We will discuss the different implementations that have arisen in the context of eHealth, specifically in the area of home healthcare, whose solutions are based on SOA.

Omar et al. [28] proposed a remote eHealth monitoring system (EHMS) for tracking patients. This system was applied in a case study where the constant monitoring of the status of pregnant women was performed. The data collected by the sensors were grouped by categories according to the quarter in which the woman was pregnant. According to the authors, EHMS uses a SOA as a model for deploying, discovering, integrating, implementing, managing and invoking eHealth services.

In [29], the design and implementation of a management system of personal health system is presented. It is divided into the following levels: devices for personal health with wireless communication capabilities (PHDs), transformation and transmission gateway (TTG), and integrated service server (ISS). The layer or TTG level is responsible for receiving readings from PHD devices, translating the messages and consuming the services offered by the ISS layer. It also consumes generated alerts, which would be created based on the readings from the PHD device.

Gnapathy et al. [6] proposed a framework based on SOA for healthcare. This SOA framework is divided in to four levels: business, data, application and technology architecture. Data mining on patient data leads to proper clinical decision, accurate visualization and monitoring of data through SOA. This implementation considers the use of several body sensors like arm cuff sensor for blood pressure monitoring and pulse oximeter sensor for determining heart rate. The medical data collected from the body sensors are routed to the sink node and data stored in the database.

Hein et al. [30] presented a system that provides health services online. One of these services is a system for tele-rehabilitation of cardiac patients after myocardial infarction. The hardware consists of a modified bicycle ergometer for training at home and a set of sensors. Different implemented services are supported by an online health infrastructure that enables the exchange of data related to the patient between different health institutions and home care systems.

A system for hypertension management of is proposed in [31]. This architecture consists of two subsystems, one that resides in the clinic and another at the site of the patient. A BASN is the subsystem located at home; data collected by the sensors are sent to the subsystem located somewhere in the clinic, where the general database of patient data is located as well as medical prescription.

Rao et al. [32] presented “e-SURAKSHAK”, a health care management system that sense vital health parameters. The sensed health parameters are communicated to a centralized electronic database wirelessly. Remote access to the monitored patients is done through standard IP protocols. The system allows a health professional to check the vital signs of a patient in a remote control room or at the patient's home. Rocha et al. [33] proposed SOCBeS framework; its main purpose is the prevention and early detection of chronic diseases through the tracking and monitoring of patients. SOCBeS collects patient data in real time and uses the cloud to store them. The SOA framework allows easy access to data by doctors or any authorized person.

Benharref et al. [34] proposed a framework to collect data from patients suffering from chronic diseases called Service Oriented and Cloud-Based eHealth System (SOCBeS). The data would be collected in real time, stored in the cloud and made available via SOA to allow easy access by physicians, paramedics, or any other authorized entity. The electronic healthcare system, was called Service Oriented and Cloud-Based eHealth System (SOCBeS). Gazzarata et al. [35] presented an open approach for clinical data interchange in cardiac tele-monitoring applications. The proposal is based on the service-oriented architecture (SOA) and was designed and developed within the CHIRON (Cyclic and person-centric Health management: Integrated appRoach for hOme, mobile and clinical eNvironments) project.

BSNCare+ was proposed in [36], it is an IoT-based health care system which implements BSN. Another application was proposed in [37], it name is BSN-Care. This proposal take into account IoT concepts. In this development the patient has body sensors of electrocardiogram, pressure in the blood among others, generating alerts on an anomaly present in a respective measure. Other important applications that implement SOA in the solution of the monitoring system are proposed in [3,38,39,40,41,42,43,44,45,46,47,48,49,50,51,52]; these do not monitor patients with a specific disease. The personal health system platform developed collects medical information, especially vital signs.

## 5. Analysis from the Perspective of Implemented Functionalities

### 5.1. Measured Variables

In the literature review, we can identify a set of parameters of interest, which are measured in the applications/frameworks studied. These parameters of interest are measured by physiological and environmental sensors. They are described below.

- Blood pressure: Pressure of the blood on the walls of the arteries as the heart pumps it around the body.
- Heart rate: Speed of the heartbeat.
- Blood sugar: Sugar concentration in the blood.
- Body temperature.
- SpO2: Oxygen saturation in the blood.
- Body composition: Related to fat, weight, and muscle mass.
- Electrocardiogram (ECG): Reading of electrical activity of the heart.
- Photoplethysmograph PPG: It is used to estimate blood flow to the skin using infrared light [53].
- Breathing rate: Number of breaths per minute.
- Acceleration: Used to detect body movements; it helps to detect abnormal behavior.
- Environmental temperature.
- Humidity.
- Dust concentration.
- Pollution.

Out of the 14 parameters found, nine come from physiological sensors, and five from environmental sensors. Sensors that allow the measurement of these parameters are described in Table 2.

It is important to note that a single device may be able to measure several parameters at once, as in the case of the solution presented in [35], while other solutions such as the one presented in [3] can use separate devices to measure each of the parameters. Table 3 shows in detail what parameters were measured in the proposed applications.

**Figure 1:**
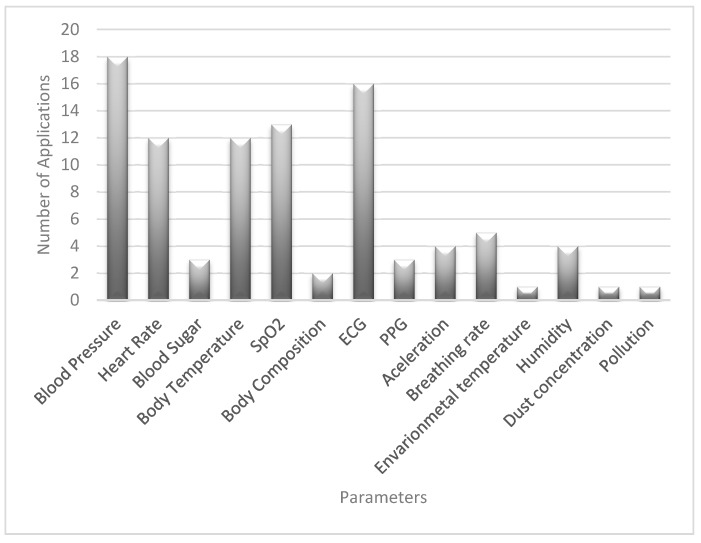
Parameter vs. applications.

In addition, Figure 1 shows the relationship between the parameters and the number of applications using these parameters. For example, blood sugar parameter was used in three different applications meaning that three of the studied proposals used this parameter in the design of BASN. The most common parameter among the proposals was blood pressure followed by ECG and heart rate; these last two have the same frequency. On the contrary, environmental parameters were less used.

**Figure 2:**
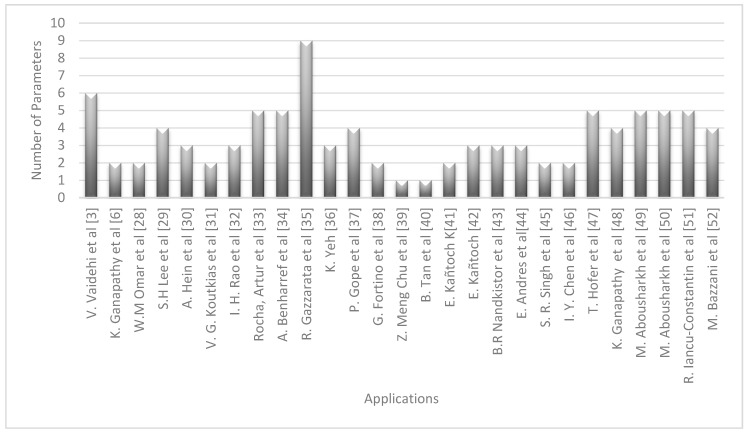
Number of parameters vs. applications.

Figure 2 shows the relationship between each of the articles reviewed and the number of variables that has been measured. An example can be the proposal made by Vaidenhi et al. [3] where six different parameters were measured. It is necessary to clarify that each proposal is related to a different application; therefore, the most complete proposal in terms of measurement of variables is presented in [35]. On the other hand, proposals with the fewest variables captured were the ones presented in [39,40].

**Figure 3:**
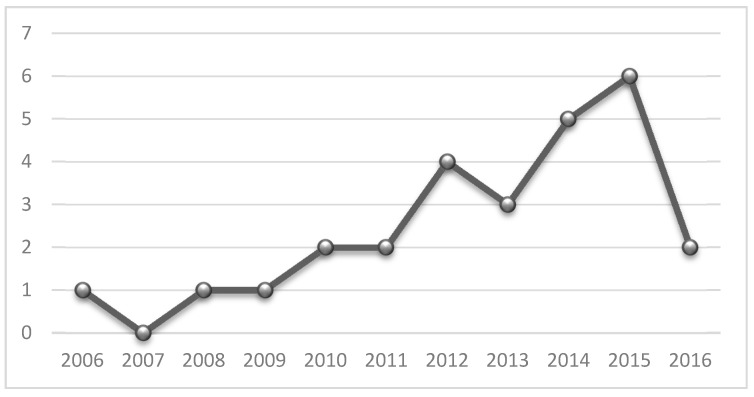
Trend by year.

Figure 3 shows research mentioned in Section 4.1 through their publication years. The articles were grouped by their publication year. For example, in the revised bibliography, we can find two articles published during 2010.

Another important point to consider is the inclusion of security in the developed proposals. Although it is true that SOA provides great benefits, it can also create multiple risks if the minimum security conditions are not taken into account. As reviewed in Section 3, and according to Cummins [20], SOA increases the number of access points for enterprise systems, thus increasing the vulnerability of the site because many of these points are exposed to the Internet. However, despite imminent risks, not all authors considered this factor. It is important that these systems maintain data integrity to achieve successful monitoring by medical professional. They similarly must maintain patient privacy and prevent unauthorized personnel gaining access to data.

**Figure 4:**
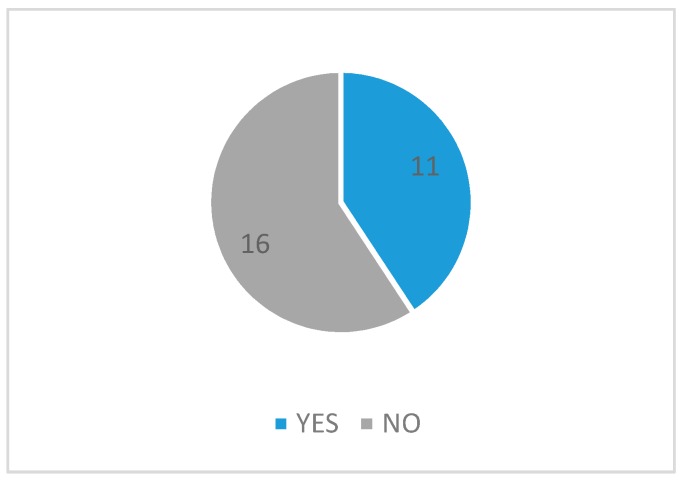
SOA applications in eHealth with and without security implementations.

Figure 4 shows SOA applications in eHealth with and without security implementations. The first category refers to the research in the state of the art in the last 10 years without security implementations, and the second one applying security processes.

According to Figure 4, only 11 applications (40.7%) implemented at least one security mechanism. Each of these mechanisms is detailed in the following section.

### 5.2. Semantic Interoperability

Semantic interoperability allows solutions to interchange data between processes and other applications in a standardized way. Semantic standardization has been present in healthcare information management for multiple years, but, in SOA healthcare application, it has been treated mainly from the research perspective.

One approach for achieving standardized semantic communication is the use of intermediate agents to standardize data exchange. For example Meo et al. [54] used in their architecture three types of agents: one for the patients, one for the healthcare service provider, and a coordinator agent. The coordinator agent service helps as a load balancer and administrator of requests, to help guaranteeing accessibility to all the services. These agents were implemented as services in a SOA solution and served the purpose of translating types of formats, which then guarantees semantic interoperability. Another interesting related project is the work by [55]. They presented a SOA solution to guarantee healthcare semantic interoperability. The key of their solution is using two types of agents. A security agent in charge of securing any data being exchanged between processes and applications, and a data interchange agent. This agent helps the solution to convert all received formats of data to a standard format that makes data retrieval and analysis easier. It uses two XML schemas: one for the service provider and one for the service consumer.

Semantic interoperability has also been addressed in SOA with generalized solutions from the perspective of IoT and pervasive computing. Kiljander et al. [56] addressed the problem by generalizing services and treating them as IoT devices, which at the same time offer services. They rely on an information centric view of the semantic interaction. They use autonomous agents introduced into the architecture, which interact by sharing semantic information. For this, they use Resource Description Framework (RDF), and Web Ontology Language (OWL). The work in [57] also focused on a generalized solution for semantic interoperability in a network of heterogeneous IoT devices. In their architecture, they use semantically annotated data that are uploaded to a new semantic mediator layer with a vocabulary builder, ontology registry and semantic engine. They use RDF to store the semantic information and the SPARQL query language to retrieve information from the RDF files.

The work in [58] also tackled the problem relying mainly on web ontologies. They built an extra semantic interoperability layer on top of the implementation layers (application, services and sensor layers). They also built a semantic architecture with different layers to link them with the applications, and data storages. One of the key components of their solution are the ontology mappers to translate semantic information in two ways, from their architecture to the implementation layers and backwards. The work in [59] presented a semantic interoperability architecture for sensor networks applications, specifically for a remote patient monitoring application. The key contribution of their work is the creation of an ontology framework to represent concepts regarding examinations as well as measurements. The idea is to correctly interpret data from heterogeneous sensors.

Semantic interoperability has also been addressed from the cloud, where most of SOA healthcare applications are stored or will be stored in the close future. The work of [60] proposed a solution using semantic web technologies to allow semantic interoperability among software agents in the cloud, with a healthcare application as a case study. They used the concept of agents with similar purposes than previously cited work. Agents represent clients and healthcare service providers. These agents provide information encoding and decoding capabilities for ontologies processing. The cloud stores the document base and serves as intermediary in the communication between the parts. Table 4 shows a comparison of implementation types and standards used by the solutions implementing semantic interoperability.

**Figure 5:**
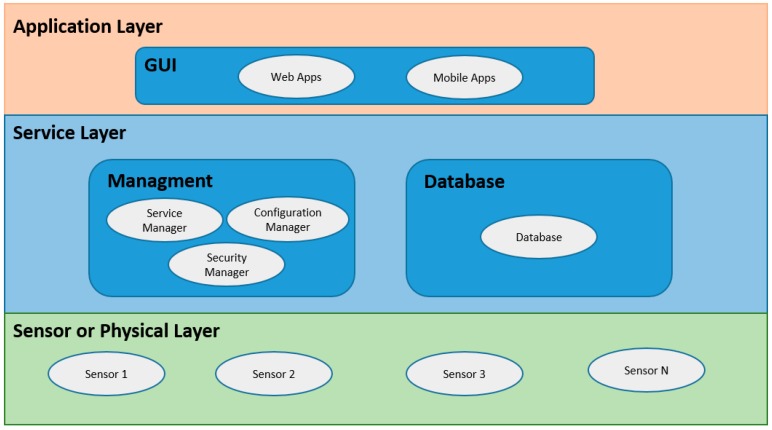
Architecture used in the revised applications.

By performing an analysis of the architectures used in the different implementations, these can be illustrated in Figure 5. In general, three layers are managed within the architecture: physical layer, service layer, and application layer. The physical layer contains the sensors, which perform the readings of the different variables of interest. The service layer can be divided into two sections: the database section and the administration section. The database section contains the management of the database of the system, and the administration section contains the management of the services necessary for the proposed application to work, these can be data processing, web services, security mechanisms, etc. The application layer contains the necessary applications to display the information by the interested user, whether patient or doctor. These can be desktop applications, web applications, or mobile applications.

## 6. Analysis from the Perspective of Security Implementations

There are different ways to implement security in SOA applications. According to [37], security is divided into two aspects: network security and data security. Network security comprises authentication, anonymity, and secure localization. However, data security includes data privacy, data integrity, and data freshness. The following are the different mechanisms used to implement security within the developed systems.

In the case of [30], the information privacy during transmission is ensured by using the DPWS implementation [61] of the WS-Security standard. The system for hypertension management proposed in [31] has mobile base units (MBUs), in which the data taken by the sensors are processed. These units provide data encryption by using an advanced standard encryption algorithm (AES) [62] which uses 128-bit keys.

On the other hand, in the case of the implementations exposed in [34,35], security was left in the hands of the HTTPS protocol. Yeh et al. [36] implemented a robust authentication system generating a random number and an exclusive function. The security in [37] is divided into two aspects: network security and data security. Network security comprises authentication, anonymity, and secure localization. On the other hand, data security includes data privacy, data integrity, and data freshness. To solve these security requirements, the authors proposed an authentication algorithm called Lightweight Anonymous Authentication Protocol.

Hofer et al. [47] used RESTful services as part of the architecture, and on top of that they built a secure REST approach, assembling authentication and authorization in a request. In the case of [48], security and privacy issues are addressed using the role based authorization model and hence improves QoS. In essence, this is an authentication module. Something similar happens with the proposal made in [49] but this time the authors do not refer to the quality of service of the network, only to the authentication of users.

In [51], the main back-end component that transfers sensitive patient data uses the cryptographic protocol Transport Layer Security (TLS). Its authentication is based on the authentication tokens and X.509 certificates. The security section in proposal [52] is focused on the implementation of SASL (Simple Authentication and Security Layer) and TLS (Transport Layer Security) protocols. Table 5 shows how security was implemented in the applications reviewed.

**Figure 6:**
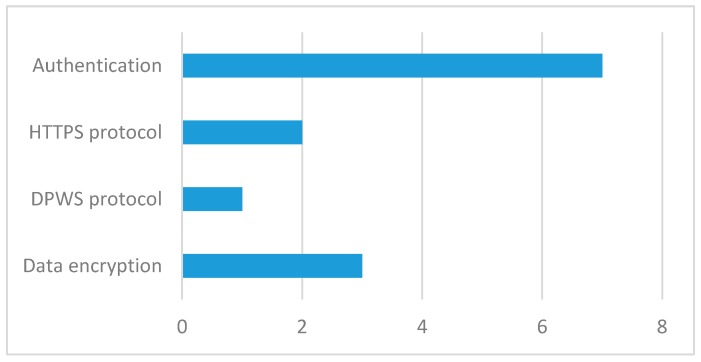
Mechanisms used to strengthen security in revised applications.

Finally, Figure 6 shows the mechanisms used to strengthen security in the revised applications. The most popular of these mechanisms is authentication with seven occurrences. It is important to note that this method is the most popular in recent years (Table 5).

## 7. Conclusions

SOA is implemented to allow seamless integration of different technologies, applications and services. We found in the proposed solutions a common architecture with two subsystems: the subsystem located in the patient’s home, and the subsystem located in the clinic or healthcare provider. The service provider in the SOA architecture can stay in the clinic or at home. In the first case, i.e., when services are offered by the clinic, the subsystem at home would consume services that would allow it to read historical data, or generate alerts for any measured parameter out of range, for example. In the second case, i.e., when the SOA services provider subsystem is stored at home, the services offered could include tasks such as querying of measured data, or verification of medicine intake.

Regarding the data being measured in these systems, it is very common to measure the patient's vital signs, including blood pressure and heart rate; such parameters are of interest to physicians to monitor in elderly people. While it is true that within these systems it is possible to find environmental sensors, it was not identified as a trend. It is important to remember that the purpose of the implementation of this type of sensor is to consider the state of the place where the patient is (light intensity, open/closed, turned on or off appliances, etc.). Regarding the security issue, it is important to find in the proposed solutions any kind of secure mechanisms to protect the system. True security can be omitted in the development of a basic prototype, but a proposed solution that reaches deployment in a production environment should be as safe as possible. It is important to remember that there is no 100%-safe level; it is the task of designers and developers to implement mechanisms to help as much as possible to increase this level of security. Semantics interoperability is an important issue that we consider has not been addressed extensively in the literature. Given the type and sensitivity of the data being stored and transmitted, we believe that in future healthcare applications based on SOA and IoT it should be considered.

The general conclusion that can be drawn is the high applicability of SOA in the systems related to health, especially in those in which the services related to information technologies are present. SOA allows a high degree of integration in heterogeneous systems, provided there is a common requirement: interoperability. SOA is considered an adequate solution for integrating heterogeneous systems, allowing application-to-application communication through the Internet.

## Figures and Tables

**Table 1:** Search results.

Key Words	Database	Results
Healthcare system using SOA	IEEE	57
	Science Direct	665
	Scopus	326
SOA and healthcare systems	IEEE	87
	Science Direct	665
	Scopus	326
Security aspects in SOA	IEEE	89
	Science Direct	1732
	Scopus	180
SOA architecture	IEEE	4688
	Science Direct	4199
	Scopus	12,985
SOA applications review	IEEE	84
	Science Direct	6366
	Scopus	162

**Table 2:** Possible devices with the measured parameter.

Category Sensor	Device	Parameters
Physiological sensor	Body scale	Body composition
	Blood pressure monitor	Blood pressure, heart rate
	Pulse oximenter	SpO2, heart rate, PPG
	Wearable vital signal monitor	Heart rate, breathing rate, body temperature
	ECG	Electrical activity of the heart
Environmental sensor	Accelerometer	Acceleration, body movements
	Environmental monitor	Environmental temperature, humidity, dust concentration, pollution

**Table 3:** Parameters measured by solutions.

Reference	Physiological Sensor	Environmental Sensor
Vaidehi et al. [3]	Blood Pressure, Heart Rate, Body Temperature, SpO2, ECG, Breathing rate	N/A
Ganapathy et al. [6]	Blood Pressure, Heart Rate	N/A
Omar et al. [28]	Blood pressure	N/A
Lee et al. [29]	SpO2, Body Composition, Blood Sugar, Blood pressure	N/A
Hein et al. [30]	Blood Pressure, SpO2, ECG	N/A
Koutkias et al. [31]	Blood Pressure, Heart Rate	N/A
Rao et al. [32]	Blood Pressure, Heart Rate, SpO2	N/A
Rocha et al. [33]	Body Temperature, SpO2, ECG, PPG	Acceleration
Benharref et al. [34]	Blood Pressure, Heart Rate, Blood Sugar, Body Temperature, SpO2	N/A
Gazzarata et al. [35]	Blood Pressure, Heart Rate, Body Temperature, SpO2, Body Composition	Environmental temperature, Humidity, Dust concentration, Pollution
Yeh [36]	ECG, Blood pressure, Temperature	Humidity
Gope et al. [37]	ECG, Blood pressure, Temperature	N/A
Fortino et al. [38]	ECG, PPG	N/A
Meng et al. [39]	N/A	Acceleration
Tan et al. [40]	N/A	Acceleration
Kañtoch [41]	ECG, PPG	N/A
Kañtoch [42]	ECG, Temperature	Humidity
Nandkistor et al. [43]	ECG, Temperature, Heart rate	N/A
Andres et al. [44]	ECG, Blood Pressure, SpO2	N/A
Singh et al. [45]	ECG	Acceleration
Chen et al. [46]	Blood Pressure, ECG	N/A
Hofer et al. [47]	Blood Pressure, Heart Rate, Body Temperature, SpO2	Acceleration
Ganapathy et al. [48]	Heart Rate, ECG, Breathing rate	Acceleration
Abousharkh et al. [49]	ECG, Blood Pressure, Body Temperature, SpO2, Heart Rate	N/A
Abousharkh et al. [50]	ECG, Blood Pressure, Body Temperature, SpO2, Heart Rate	N/A
Iancu-Constantin et al. [51]	ECG, Blood Pressure, SpO2, Heart Rate	Acceleration
Bazzani et al. [52]	Blood Pressure, Blood Sugar, Body Temperature, SpO2	N/A

**Table 4:** Architectures and standards used in semantic interoperability solutions.

Reference	Type of Solution	Standards Used
[54]	2 types of intermediate agents	Not defined
[55]	3 types of intermediate agents	XML
[56]	Mid-tier agents	RDF, OWL
[57]	Ontology mappers	OWL
[58]	Semantic mediator layer	RDF, SPARQL
[59]	Ontology to represent concepts and data	XML
[60]	Ontology decoders and encoders based on cloud	XML, RDF

**Table 5:** Security implementations in SOA health applications.

Reference	Reference Year	How Security Was Implemented?
Hein et al. [30]	2009	Data encryption
Koutkias et al. [31]	2010	DPWS protocol
Benharref et al. [34]	2014	HTTPS
Gazzarata et al. [35]	2014	HTTPS
Yeh [36]	2016	Login
Gope et al. [37]	2016	Login
Hofer et al. [47]	2015	Login
Ganapathy et al. [48]	2013	Login
Abousharkh et al. [49]	2012	Login
Iancu-Constantin et al. [51]	2015	Data encryption, login
Bazzani et al. [52]	2012	Data encryption, login
